# Hospital readmissions in late preterm infants

**DOI:** 10.1186/1824-7288-40-S2-A29

**Published:** 2014-10-09

**Authors:** Marco Pezzati

**Affiliations:** 1 Neonatology and NICU, Ospedale San Giovanni di Dio, ASL 10 Firenze, Italy

## 

Neonatologists generally recognize that late preterm infants face more problems in the immediate newborn period compared with their full-term counterparts. [1,2]. This excess morbidity extends beyond the initial birth hospitalization [3] and the literature recognizes that readmission rates of late preterm infants are 1.5 to 3 times that of term infants [4-7]. In this group of infants, the overwhelming reasons for rehospitalisation are jaundice and feeding problems.

The most widely studied metric of health care utilization in late preterm infants is short-term readmission (the first two weeks) after birth hospitalization. Escobar [8] found that late preterm babies with short NICU stays had the highest rehospitalisation rates and in a follow-up study they found that rehospitalisation rates within 2 weeks were higher among late preterm infants who never were admitted to the NICU [4]. Shapiro-Mendoza [7] found that late preterm babies discharged early were at greater risk of neonatal morbidity. They also found that risk factors for subsequent readmissions were birth hospital stays less than 4 days, breastfeeding, Asian/Pacific Islanders, first born infants, and public payers at the time of delivery. In the United Kingdom, Oddie [9] also noted that late preterm infants had the highest rate of readmission but infectious disease and not jaundice was the leading factor for readmission, which the investigators attributed to a differing approach to management of jaundice in the United Kingdom.

Escobar[10] examined late rehospitalisation (after the first two weeks) and found 36 week gestation newborns at higher risk for readmission. Paradoxically, babies of 34 and 35 weeks were not at higher risk and this may be explained by more frequently delayed discharge of infants of shorter gestational age. McLaurin [5] also demonstrated increased late rehospitalisation rates in late preterm infants and found that the subset with prolonged birth hospitalizations (≥4 days) had the highest rates of rehospitalisation. Respiratory disease (bronchiolitis and pneumonia) was the most common cause of readmission.

The late preterm infant is particularly responsive to the benefits but vulnerable to the risks of early discharge home. Longer length of stay before discharge is protective against readmission but it is not reasonable to prolong the birth hospitalization of newborns who meet criteria for discharge. More effort needs to be placed to reduce the risk of jaundice and feeding problems in these patients: to avoid mother and infant separation during birth hospitalization, to arrange a follow-up appointment within 48 hours of discharge, to promote and support lactation before and after discharge.

## Conflict of interest

The author has no conflict of interest to declare.
